# Case series: expanding diagnostic markers in postorgasmic illness syndrome

**DOI:** 10.1093/sexmed/qfac021

**Published:** 2023-03-02

**Authors:** Leah Rosetti, Amin Kanani, Luke Witherspoon, Ryan Flannigan, Stacy Elliott

**Affiliations:** Department of Psychiatry, University of British Columbia, 255 Wesbrook Mall, Vancouver, BC V6T 2A1, Canada; Division of Allergy and Immunology, Department of Medicine, University of British Columbia, St. Paul's Hospital, 1081 Burrard Street, Room 166, Vancouver, BC V6Z 1Y6, Canada; Department of Urologic Sciences, University of British Columbia, Vancouver General Hospital, Level 6, 2775 Laurel Street, Vancouver, BC V5Z 1M9, Canada; Department of Urology, The Ottawa Hospital - General Campus, 501 Smyth Road, Ottawa, BC K1H 8L6, Canada; Department of Urologic Sciences, University of British Columbia, Vancouver General Hospital, Level 6, 2775 Laurel Street, Vancouver, BC V5Z 1M9, Canada; Department of Urology, Weill Cornell Medicine, 525 East 68th Street, Starr 9, New York, NY 10065, United States; Department of Psychiatry, University of British Columbia, 255 Wesbrook Mall, Vancouver, BC V6T 2A1, Canada; Department of Urologic Sciences, University of British Columbia, Vancouver General Hospital, Level 6, 2775 Laurel Street, Vancouver, BC V5Z 1M9, Canada; GF Strong Rehabilitation Centre, 4255 Laurel St, Vancouver, BC V5Z 2G9, Canada

**Keywords:** Postorgasmic illness syndrome, POIS, ejaculation, case series

## Abstract

**Introduction:**

Postorgasmic illness syndrome (POIS) is a rare condition in which individuals develop generalized and flu-like symptoms after ejaculation. Several mechanisms and treatments for this disorder have been proposed but many questions remain.

**Aims:**

We sought to present a summary of literature to date and highlight common symptoms, associated features, comorbidities, and laboratory findings in a clinical sample of patients with POIS.

**Methods:**

We conducted a retrospective chart review of 6 patients with POIS in our clinic and presented compiled results.

**Results:**

We identified newly described non–flu-like presentations, onset of symptoms with high states of arousal without ejaculation, and presence of psychiatric comorbidity in a majority of patients. We did not identify a postorgasm allergic response with bloodwork available.

**Conclusion:**

POIS remains a poorly understood condition that likely comprises a number of different clinical entities. Further research on a larger clinical sample is necessary to better characterize POIS and understand its biological and psychological basis.

## Introduction

Although postorgasmic illness (POIS) was first described more than 20 years ago, data regarding its prevalence and etiology remain elusive. The lack of clarity in relation to the pathophysiological underpinnings of this condition has precluded the development of effective targeted treatments.^1^ Despite emerging literature on POIS, there remains a lack of awareness of the condition.^2^ Patients may be hesitant to present to their physician with their unusual symptoms associated with sexual activity—thereby making it difficult to gather patient data to better define the illness.

Symptoms of POIS vary widely, with the most common symptoms being fatigue, irritability, and concentration difficulties.^3^ In one case series of men with POIS, symptoms were acknowledged after most ejaculations following masturbation (95.7%), intercourse (77.0%), and nocturnal emission (57.4%).^4^ Symptoms appear to vary in relation to the time of onset—with a majority occurring within 30 minutes of ejaculation, peaking in 12-48 hours, and lasting for 2-7 days.^4^

In this paper we present the summary of 6 cases of POIS, highlighting newly described symptoms occurring postorgasmically, psychiatric comorbidities, and occurrence of symptoms with high states of arousal, which to our knowledge have not previously been described in the literature and may provide clues as to the etiology of this condition.

## Materials and methods

We evaluated 6 male patients seen for medical care in our clinical program who have received a diagnosis of POIS. This diagnosis was made using the criteria for POIS derived from Waldinger.^5^ These patients were not actively recruited but were referred to our clinic for evaluation of issues related to postorgasmic symptoms. We conducted interviews for routine clinical care in addition to obtaining historical information via a retrospective chart review, for which ethics approval was obtained through our institutional research ethics board. A scoping review was conducted, and the most recent and relevant literature has been cited.

## Results

The mean age of subjects at time of assessment was 32 ± 5.4 years. Of the 6 male subjects, all were heterosexual. Three of these patients were in relationships and the remainder were single. All subjects were employed. Three of our 6 patients had a history of depression or anxiety, possibly suggesting shared vulnerability. Four of our patients had environmental allergies, consistent with findings of other studies. Further comorbidities are outlined in Table 1. No patients reported a family history of similar symptoms.

**Table 1:** Comorbidity and POIS chara cteristics in 6 patients.

**Patient history**	**Patients**					
	**A**	**B**	**C**	**D**	**E**	**F**
Psychiatric comorbidity						
History of anxiety disorder		✓	✓	✓		
History of depressive disorder		✓	✓		✓	
Other psychiatric symptoms			✓			
Medical comorbidity						
Asthma				✓		
Eczema	✓					
Allergies (environmental/food/drug)		✓	✓	✓	✓	✓
Urinary symptoms	✓	✓				
Migraines			✓			
Comorbid sexual dysfunction						
Premature ejaculation		✓				
Erectile dysfunction			✓		✓	
Low libido			✓		✓	
Clusters						
General						
Extreme fatigue	✓	✓	✓	✓	✓	✓
Exhaustion			✓	✓	✓	✓
Word-finding difficulties			✓	✓	✓	
Concentration difficulties			✓	✓	✓	✓
Irritablility			✓	✓	✓	✓
Photophobia			✓	✓		✓
Depressed mood			✓	✓	✓	✓
Flu Like						
Feeling cold	✓	✓		✓		
Head						
Headache		✓			✓	✓
Foggy feeling in head			✓	✓	✓	✓
Heavy feeling in head			✓		✓	✓
Throat						
Sore throat		✓	✓		✓	✓
Muscle						
Tension behind neck				✓	✓	✓
Muscle weakness		✓	✓	✓	✓	✓
Heavy legs		✓	✓	✓	✓	
Stiff muscles		✓			✓	✓
Uncategorized						
Increased hunger			✓	✓	✓	✓
Cold intolerance	✓		✓	✓		
Enlarged lymph nodes		✓			✓	✓
Memory challenges			✓	✓	✓	✓
Symptom characteristics						
Immediate onset after ejaculation	✓	✓	✓	✓	✓	✓
Time to resolution						
<1 week	✓			✓	✓	✓
>1 week		✓				
Variable			✓			
Situation						
Occur with self-stimulation	✓	✓	✓	✓	✓	✓
Occur with nocturnal emission^a^	✓	NA	✓	✓	NA	NA
Occur with partner^b^	✓	✓	✓	✓	✓	NA
Occur with every ejaculation	✓	✓	✓	✓	✓	✓
Occur with high states of arousal						
Yes		✓	✓			
No	✓			✓	✓	✓
Dose-dependent response						
Yes		✓		✓		✓
Unclear			✓		✓	
Age of onset						
Puberty	✓	✓				
Later in life			✓	✓	✓	✓

a Only 3 of the patients experienced nocturnal emission.

b 1 of the patients has not yet had partnered intercourse.

All of the patients in our group experienced symptoms immediately after ejaculation, with every ejaculation, whether nocturnal emission or self- or partnered sexual stimulation. The proposed criteria for POIS suggest that symptoms can last for up to 7 days after ejaculation, but in a follow-up study of the validity of those criteria the investigators noted that symptoms occurred in some men for periods longer than 7 days,^3^ which was the case for 2 men in our sample.

The sites of the most commonly represented symptom clusters in our small group of patients were the head, general, and muscle symptoms, consistent with findings in larger samples.^3^ All 6 of our patients reported extreme fatigue after ejaculation, and 5 of 6 patients had muscle weakness. Half of our patients (3 of 6) described previously unreported postorgasm symptoms of increased hunger, cold intolerance, enlarged lymph nodes, and memory challenges.

Baseline physical exam results were unremarkable. Laboratory investigations in our group were not consistent or available across cases, in part due to the inconvenience of seeking a postorgasm blood sample. However, only 1 of our patients had relatively low concentrations of testosterone and FSH (follicle-stimulating hormone), and we did not uncover any clear derangements in the other bloodwork that would point to etiology. Three patients had normal serum levels and C-reactive protein (CRP) postorgasm.

Perhaps most significantly, 3 of our 6 patients described onset of POIS-like symptoms occurring with high states of arousal without ejaculation, possibly suggesting involvement of pre-ejaculate, which may contain small amounts of sperm, or sympathetic autonomic recruitment seen with seminal emission.

## Discussion

Cases of POIS have historically been defined using the criteria proposed by Waldinger, which have been validated in 1 study drawing from subjects in an online forum.^3^ Despite increasing discussion of POIS in the literature, its etiology remains poorly understood and may represent a number of different conditions.

An immunological response to components of seminal fluid could contribute to the pathogenesis of POIS. It was initially proposed that POIS was IgE mediated, given the rapid symptom onset after ejaculation and systemic nature of symptoms, supported by reduction in symptoms by hyposensitization treatment with autologous semen in 2 individuals.^6^ Presence of symptoms following surgical sterilization led to theories that the antigen was present in seminal fluid.^5^ The role of IgE and a T helper type 2 (TH2) immune response in POIS has since been brought into question, demonstrated by positive skin-prick testing to autologous semen in controls and negative IgE in vitro tests in individuals with positive skin-prick tests.^7^ This is supported by our finding in 1 case of positive skin-prick reaction to autologous semen in the patient as well as his wife. In addition, the serum tryptase level was not elevated postorgasm in 3 of our cases, suggesting there was no mast cell activation, a key feature of IgE-mediated allergic reactions.

The role of the adaptive immune response in POIS is poorly understood. Even less understood is whether there is an innate immune response contributing to the pathogenesis of this disorder. In 3 of the cases presented, the CRP level was normal postorgasm. This brings into question whether POIS is due to an inflammatory response.

Other proposed mechanisms, including involvement of the COX (cyclooxygenase) pathways, comparative deficiency in opioids following ejaculation superimposed on psychological risk factors, and postorgasmic autonomic hyperactivity, demonstrate the lack of consensus about the physiologic basis of these symptoms.^7^

With regard to comorbidity**,** in 1 cohort, 58% of men with POIS reported having various forms of allergies, suggestive of atopic constitution.^6^ This finding is consistent with our sample, in which 4 of 6 patients had environmental allergies, although none of our patients had a response to antihistamines or benefit from immunotherapy, decreasing the likelihood that our patients had an allergic component to their presentation.

A recent study revealed that the most commonly reported comorbidities with POIS were premature ejaculation (46.0%), depression (24.3%), and generalized anxiety disorder (17.9%).^4^ Three of our 6 patients had a history of depression or anxiety, higher rates than would be expected in the general population. We recognize that there is complex interplay between medical symptoms and mood and that this area requires further investigation into the directionality of the relationship. There is overlap between depression, anxiety, and somatization,^8^ which suggests the possibility that some individuals with POIS, especially those who have inconsistent or physiologically implausible symptoms, have a component of somatic symptom disorder that could be a fruitful target for treatment. Individuals with depression and anxiety could also be more sensitive to changes in endogenous opioids following orgasm.

One of our patients presented with interstitial cystitis and IBS (irritable bowel syndrome), in addition to POIS. Though this is only 1 patient, we feel that it is possible that in a subset of POIS patients, at least a portion of their POIS symptoms (most likely pain-related symptoms) are mediated by central sensitization, in which the nervous system is regulated to be in a persistent state of high reactivity.

A variety of POIS treatments have been outlined in the literature, with variable and often limited effects, including antihistamines, SSRIs (selective serotonin reuptake inhibitors), NSAIDs, silodisin, immunotherapy, psychotherapy (flooding), and nutritional supplements.^7^^,^^9^ None of these treatments were consistently effective in our patient group. Niacin provided some benefit in 2 of our patients, but this effect was short lived.

Interestingly, 2 of our patients described behavioral management of their symptoms by uncoupling orgasm and ejaculation, a noninvasive option requiring motivated training. This treatment was effective in allowing these patients to enjoy sexual activity with their partners without POIS symptoms, something not previously described. Fortunately, neither of these patients were pursuing fertility.

Recently, 2 case reports outlined targeted treatment of symptoms with different agents based on symptom cluster. Symptoms similar to those of opioid withdrawal were treated with tramadol, and allergy-type symptoms were treated with antihistamines with good effect.^10^ It is likely that the diagnosis of POIS captures a variety of conditions with different mechanisms, and we advocate for treatment targeting both physical and psychological substrates, paying attention to individual variations in symptom profiles when choosing treatment.

Our sample was too small to be statistically analyzed for patterns emerging between POIS symptoms, characteristics, and comorbidity. Therefore, this series is only descriptive in nature, creating an opportunity for further research with larger sample sizes.

## Conclusion

POIS is a rare condition which at this point remains poorly understood. Our case series highlights newly described medical signs and symptoms, psychiatric comorbidity, and presence of symptoms with high states of arousal in a small sample of men (*n* = 6). Three of our patients in this series experienced POIS symptoms without ejaculating, possibly pointing to involvement of pre-ejaculate as a causative factor in some cases. Additionally, we highlight the presence newly described postorgasmic symptoms occurring in 3 of our 6 patients including increased hunger, cold intolerance, enlarged lymph nodes, and memory challenges. We also question whether there is an allergic or inflammatory process causing this condition, with normal tryptase and CRP postorgasm in 3 of our cases. Those patients who could decouple orgasm and ejaculation avoided symptoms, but otherwise our patients remained symptomatic at the time of submission. Overall, we agree that further research on a larger clinical sample is necessary to better characterize POIS and understand its biological and psychological underpinnings. In the meantime, sensitive and biopsychosocially informed interviews are important in validating the concerns of these patients and alleviating their distress.

## Funding

None declared.

*Conflicts of interest:* None declared.
